# Is facial pattern a predisposing factor for otitis media with effusion in children?

**DOI:** 10.1590/S1808-86942011000100014

**Published:** 2015-10-19

**Authors:** Cláudio de Góis Nery, Fernando Stefanato Buranello, Cícero Pereira, Renata Cantisani Di Francesco

**Affiliations:** 1 PhD in Sciences, Assistant Professor - Department of Orthodontics - UFG; 2 MSc, Professor Coordinator of the Orthodontics Program; 3 PhD in Experimental Social Psychology. Epidemiology and Statstcs Consultant - UFG; 4 PhD in Medicine; Professor at the Department of Otolaryngology of the Medical School of the University of São Paulo - FMUSP

**Keywords:** otitis media with effusion, eustachian tube, middle ear ventilation

## Abstract

Abnormalities in craniofacial morphology are associated with Eustachian tube dysfunction and otitis media with effusion (OME).

**Aim:** to evaluate the relationship between facial pattern and craniofacial growth direction, and OME in children with enlarged tonsils and adenoids (ETA).

**Methods:** Clinical prospective survey in 79 children (41 male and 38 female), ranging from 4 to 10 years of age, with tonsil and adenoid enlargement (Brodsky's grades III and IV). Forty children presented with OME (study group) and 39 did not (control group). Cephalometric analysis was used to determine the facial pattern.

**Results:** There was no correlation observed between facial pattern and OME (c 2 = 0.25 *p* = 0.88). Facial Axis was larger in the OME group (F(1.75) = 3.68 *p* = 0.05) and the Lower Anterior Facial height was smaller (F(1. 75) = 3.99 *p* = 0.05) in children with otitis media with effusion.

**Conclusions:** There was no correlation between OME and facial pattern in children with ETA although a more horizontal facial growth direction, and a smaller lower anterior facial height was observed consistently among subjects in this group. This suggests that abnormal positioning of the eustachian tube influences the development of OME in children with ETA.

## INTRODUCTION

Abnormalities in the craniofacial morphology are associated with Eustachian tube dysfunction and otitis media with effusion (OME)^1^. Pautow reported that the Eustachian Tube morphology is associated with the shape of the head^2^. Brachyfacial adult individuals tend to have narrower tubes, and consequently more otitis. These results corroborate the statement of other authors who reported that otitis media is common in Brachyfacial patients^3,^^4^.

One of the most frequent conditions associated with OME is enlarged pharyngeal and palatine tonsills^5^.

Nonetheless, not all children in school age with enlarged pharyngeal and palatine tonsils develop OME^6^.

Based on these statements, it is possible to propose the hypothesis that the head's shape would be a predisposing factor to OME.

The goal of the present study was to establish whether or not there are associations between the facial pattern and OME in children with enlarged pharyngeal and palatine tonsils.

### Sample

This study was approved by the Ethics in Research Committee analyzing research projects - CAPPesq from the Clinical Board of the Medical School of the University of São Paulo, under protocol # 738/04.

In this clinical, prospective, case-control study we had 79 children (41 boys and 38 girls), aged between 4 and 10 years, being treated at the ENT department of the Medical School of the University of São Paulo.

All the patients had chronic upper airway obstruction because of enlarged pharyngeal and palatine tonsils. They were randomly selected from the patients' waiting list for adenotonsillectomy. 40 patients with OME and indication for ventilation tubes (study group) and 39 individuals without such indication (control group). All the individuals had at least 80% of nasopharyngeal obstruction caused by enlarged pharyngeal tonsils, established by the side x-ray taken. We included only those patients with palatine tonsil enlargement in grades III-IV, from the Brodsky classification^7^.

The study group was made up of 40 individuals who had had OME for more than 3 months, with air-bone gaps of at least 20 dB, no stapes reflex and flat tympanometry (type B). Thirty-nine individuals selected for the control group did not have OME diagnosed during physical exam (otoscopy) and confirmed by normal audiometry and tympanometry. The waiting list in this institution for adenotonsillectomy, with or without a ventilation tube, varied between 8 and 10 months. Usually the indications for a ventilation tube in this department follow the guidelines from the American Academy of Otorhinolaryngology concerning the insertion of ventilation tubes. The recommendation to insert a ventilation tube is a valid indicator in auditory tube dysfunction. The exclusion criteria were: personal or family history of cleft palate, or another craniofacial abnormality; chronic medical conditions; prior oral, nasal, pharyngeal or craniofacial surgery; prior or current orthodontic treatment. Syndromic patients with our without neurological disorders were taken off.

## METHODS

**Figure 1:**
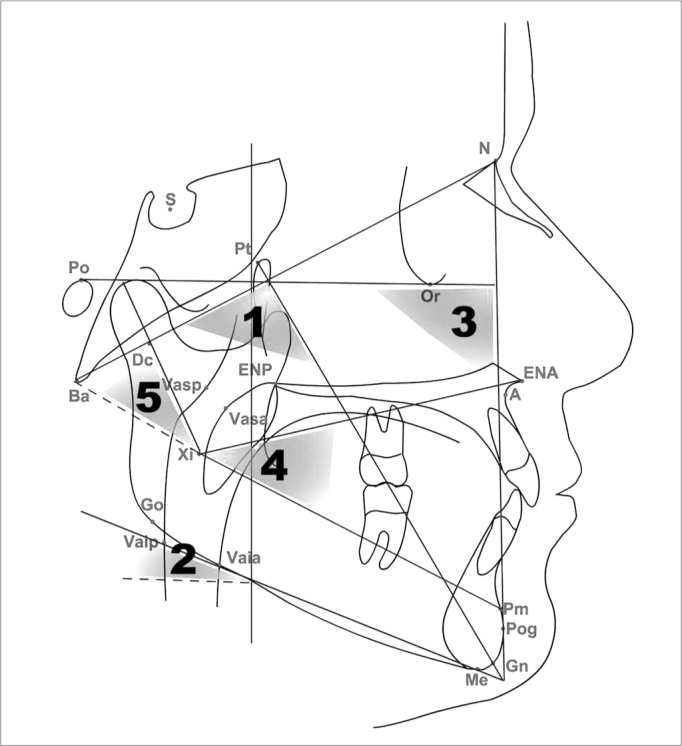
Cephalometric analysis.

All the individuals were submitted to otolaryngological anamnesis, physical exam and side X-Ray. The radiographs were taken from each individual positioned in the cephalostate with the mandible in centric occlusion and the lips at rest. All the radiographies were digitalized and the cephalometric points were identified (Table 1). We used the Radiocef 2003, 5th edition software in order to do our analysis. The cephalometric analysis was made up exclusively from the angular measures used to establish facial typology according with Ricketts et al.^8^. The VERT index (vertical growth coefficient) is determined by means of an arithmetic average of the difference between the angles and their normal values^8^. Five factors are employed in establishing the VERT index: (1) facial axis (EF), (2) facial depth (PF), (3) mandible plane angle (PM), (4) inferior facial height (AFI), and (5) mandible arch (AM) (Figure 1). After acquisition of the cephalometric data, each factor was compared to its respective standard deviation, and to this difference we assigned a positive or negative sign to indicate direction as brachycephalic or dolicofacial. Each value was broken down by its respective standard deviation, the results from the five factors are submitted to the simple average and the result determines the VERT index, which will be compared to reference values (Table 1) in order to determine the facial pattern.

**Table 1:** Established cephalometric landmarks.

N- Nasion	Me- Menton	
Or- Orbitale	Pog- Pogonion	Vasa- anterior superior airway
S- Sella turcica	Gn- Gnathion	Vasp- posterior superior airway
Po- Porion	ENA- anterior nasal spine	Vaia- anterior inferior airway
Ba- Basion	ENP- posterior nasal spine	Vaip- posterior inferior airway
Dc- Center of the condyle	Go- Gonion	A- maxillary concavity
Xi- Center of the mandible	Pt- Pterigomaxillary	Pm- mentonian protuberance

The descriptive statistical analyses (mean ± standard deviation) were calculated from the values observed for each factor. The mean difference for independent groups was calculated using the t Student test, *p* values <0.05 were considered statistically significant. Correlations between the variables were made by means of the chi-square and Person's test.

The data was analyzed using the Statistical Package for Social Sciences (SPSS for Windows 12.0; SPSS Inc. Chicago, Illinois, USA).

## RESULTS

There was no relation between facial shape and otitis media with effusion (Table 2). Based on the angular measures, we noticed that the Facial Axis value, which determines mandible growth direction, was significantly larger, and the Inferior Facial Height value was significantly smaller in the OME group (Table 3), when compared to the control group.

**Table 2:** Guide for interpreting the VERT index.

Severe Dolicofacial	Dolicofacial	Mild Dolicofacial	Mesofacial	Brachyfacial	Severe Brachyfacial
-2,0	-1,0	-0,5	0	+0,5	+1,0

**Table 3:** Comparison of the angular values in children with OME and controls.

	Groups									
Angular values	OME (^o^)	SD	n	controls(^o^)	SD	n	Norma	SD	n	*p*
Inferior facial height (Xi-ENA·Xi-Pm)	46,93	3,71	40	48,10	3,78	39	47,51	3,77	79	=,05
Facial axis (Ba-N·Pt-Gn)	85,99	3,55	40	84,73	3,49	39	85,37	3,56	79	=,05
Mandibular plane (Po-Or·Go-Me)	29,28	4,91	40	30,33	4,57	39	29,80	4,74	79	=,16
Mandibular arch (Xi-Pm·Xi-Dc)	20,55	7,62	40	20,55	8,16	39	20,55	7,84	79	=,88
Facial depth (Po-Or·N-Pog)	86,39	3,35	40	85,86	3,98	39	86,12	3,66	79	=,37

n = number of participants

(o) = angle

*p* = Student t test

SD = standard deviation

## DISCUSSION

We know that OME is more common in children because of the very characteristics of their Eustachian Tubes. During this stage of life, the tube is described as being more horizontal, in relation to the skull base, and narrower in diameter than it is in adults^9^.

More than half of the individuals in this study, including the control group had dolicofacial type. In other Brazilian studies, the dolicofacial type was also the predominant type^4,^^10^. The correlation between facial types and OME was not found in this study. Despite the prevalence of dolicofacial individuals in this study, there was a mild predominance of the brachyfacial pattern in the otitis group when compared to the control group. This finding is supported by other authors, who have also found a higher incidence of otitis media in the brachyfacial face pattern^3,^^4,^^11^. Other studies found different results^12,^^13^, with a vertical facial pattern (dolicofacial) seen in individuals with Eustachian Tube dysfunction. However, in those studies the enlargement of pharyngeal and palatine tonsils was not determined, and this may have influenced the results, as well as the sample itself. Pharyngeal obstruction and consequent oral breathing result in a more vertical facial growth in genetically predisposed individuals^6,^^14^.

The facial shape is genetically defined, because it depends on skull base shape. In this study, the sample was entirely made up of individuals with enlarged pharyngeal and palatine tonsils, with the goal of maintaining consistency among the individuals. The Facial Axis angular value was larger, while the Inferior Facial Height was lower in the OME group when compared to the control group. The facial axis describes the mandible position and, consequently, the facial development direction. It is known that the face develops in the forward and downward directions^9^, corroborating the findings of our previous study^1^. Higher Facial Axis values are associated with smaller faces. Similarly, a reduced inferior facial height may also result in a more horizontal facial growth pattern. The cartilaginous portion of the Eustachian Tube is located in the petrous-squamous portion of the temporal bone, located near the occipital and sphenoid bones^15,^ ^16^. These bones are part of the skull base and their proximity to the temporal bone explains the relationship between the head shape and the auditory tube. The skull base is the key for the growth and development of the facial structures, and its morphology can influence adjacent structures. This is known as the counterpart theory, in which the skull base determines the size of the maxilla and that of the mandible^15^. The auditory tube extends from the skull base (bone portion) all the way to the pharynx and its growth is associated with the development of the skull base and the nasomaxillary complex^17^.

The peritubal muscles (soft palate elevator and tensor muscles) go from the skull base all the way to the hard palate and cartilaginous portion of the auditory tube in the pharyngeal wall. The soft palate tensor insertion is more superior and distal to the lumen in adults when compared to children^18^. Shibahara and Sando (1988) reported that the angular insertion of these muscles can change the traction vector during tube opening and may also contribute to its dysfunction, predisposing the patient to otitis media^19^.

## CONCLUSION

Based on the data hereby presented, we can conclude that there is no correlation between OME and the facial shape in children with enlarged pharyngeal and palatine tonsils, despite a mild predominance of the brachyfacial pattern in the group with otitis. The children with OME had a more horizontal facial shape, with a reduced facial height. These findings suggest that besides the enlargement of pharyngeal and palatine tonsils, Eustachian Tube dysfunction is also associated with craniofacial morphology. Any change in craniofacial growth direction may reduce Eustachian Tube function.
